# Assessment and management of undifferentiated carcinoma with osteoclastic like giant cells of the pancreas: a case report and revision of literature

**DOI:** 10.1186/s12876-021-01779-5

**Published:** 2021-06-02

**Authors:** Elisabetta Cavalcanti, Nicolo’ Schena, Grazia Serino, Giulio Lantone, Raffaele Armentano

**Affiliations:** 1 Histopathology Unit of National Institute of Gastroenterology “S. de Bellis,” Research Hospital, Via Turi 27, 70013 Castellana Grotte, Bari, Italy; 2 Laboratory of Experimental Immunopathology, National Institute of Gastroenterology “S. de Bellis,” Research Hospital, Castellana Grotte, Bari, Italy; 3 Surgery Unit of National Institute of Gastroenterology “S. de Bellis,” Research Hospital, Castellana Grotte, Bari, Italy

**Keywords:** Pancreas, Pancreatic cancer, Undifferentiated pancreatic cancer, Giant cell tumor

## Abstract

**Background:**

Undifferentiated carcinoma with osteoclast-like giant cells (UCOGCs) is a rare and aggressive non endocrine pancreatic carcinoma characterized by the presence of osteoclastic giant cells mixed with mononuclear cell. Very few cases have been reported in the literature and the histogenesis is controversial as, at the time of diagnosis, the tumor is often of advanced size and stage and it is difficult to pathologically observe its relationship with the pancreatic duct.

**Case presentation:**

We present a case of 65-year-old male patient presenting with abdominal pain, nausea, and weight loss, which was treated with surgical resection. Histological examination revealed an undifferentiated pancreatic carcinoma with osteoclast-like giant cells. The patient underwent to a routine pylorus preserving pancreatoduodenectomy**.** Actually, the patient was in good performance status and disease-free five months.

**Conclusions:**

Based on the present case and limited previous data, further researches preferably with large cohorts are necessary to clarify the pathogenesis of the neoplasm. However, as show in this case, histopathological and immunohistochemically studies are the gold standard for the diagnosis of UCPOGC. Investigation of the genomic alterations in UPOGCs could help to explain the histologic diversity of variant tumor and could provide a genetic basis for prognosis and treatment.

## Background

Undifferentiated carcinoma with osteoclast-like giant cells (UCOGCs) is a rare and aggressive non endocrine pancreatic carcinoma characterized by the presence of osteoclastic giant cells mixed with mononuclear cell [1]. In the recent WHO Classification, UCOGCs was recognized as a morphologically and clinically distinct variant of pancreatic ductal adenocarcinoma (PDAC) [2]**.** It accounts less than 1% of all pancreatic malignancies and shows worse prognosis than invasive ductal adenocarcinoma of the pancreas and despite active intervention, patients usually die within months of diagnosis [3]. Therefore, very few cases have been reported in the literature and the histogenesis is controversial as, at the time of diagnosis, the tumor is often of advanced size and stage [4] and it is difficult to pathologically observe its relationship with the pancreatic duct. Some authors support an epithelial origin from acinar cells or ductal cells and others a mesenchymal origin [5, 6]. Joo et al. [7] reported that the giant cell tumor of the pancreas presents as two different phenotypes: undifferentiated carcinoma with osteoclast-like giant cells (OGCs) (which is highly malignant and has a poor prognosis) and a pure OGC tumor (which has a better prognosis). Undifferentiated carcinoma of the pancreas with OGCs is characterized by a dual component of undifferentiated carcinoma cells (neoplastic mono- or multinucleated cells) and multinucleated OGCs, mimicking giant cell tumor of bone. The origin of these two components within a tumor has long been discussed based on the immunohistochemical and ultrastructural analysis after pancreaticoduodenectomy. This case supports the ductal epithelial origin of undifferentiated carcinoma with OGCs and prompt diagnosis could outcome in favorable surgical outcomes.

## Case presentation

**Fig. 1:**
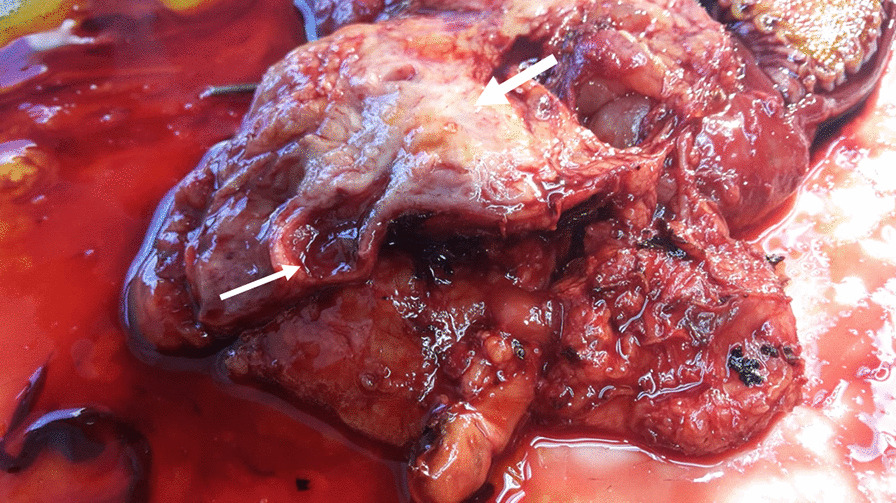
Macroscopic view of the lesion infiltrating the head of the pancreas, which encompasses the main biliary tract (big arrow): the tumor is in the head of the pancreas with a size of 3 cm. Small arrow: bile duct dilation

**Fig. 2:**
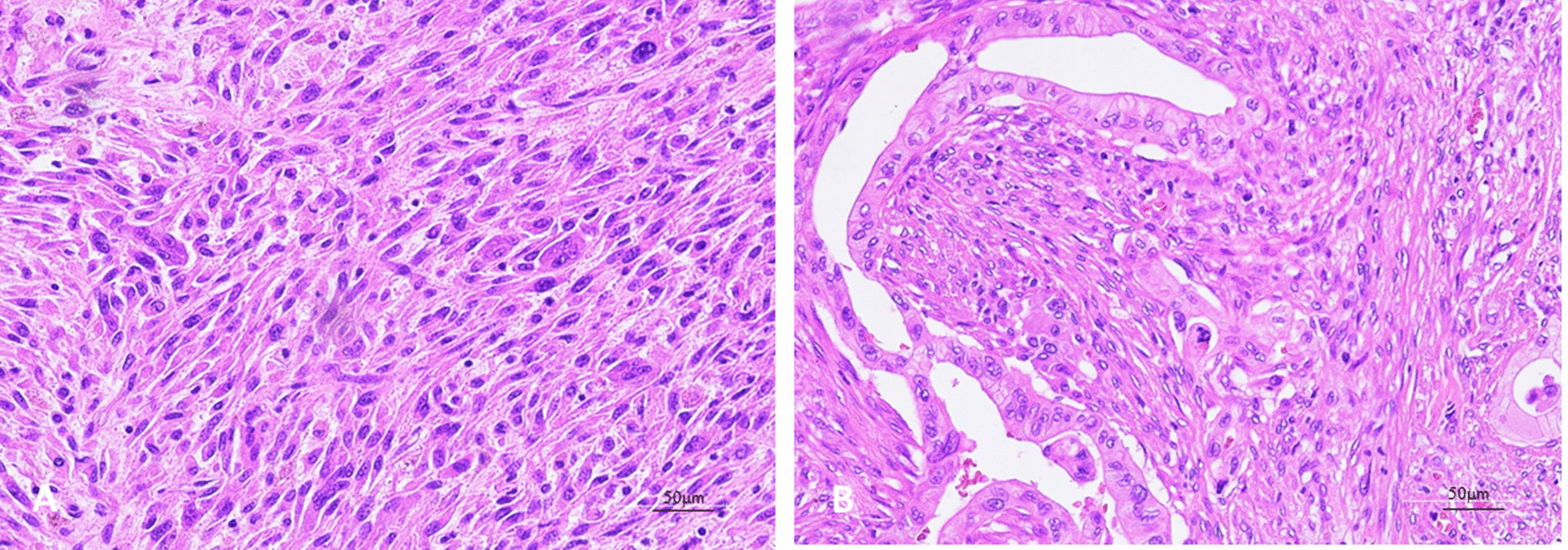
Histomorphopathologic findings **a** Undifferentiated cells with highly pleomorphism and nuclear atypia mixed among scattered osteoclast-like giant cells, **b** presence of very early ductal adenocarcinoma lesions associated and detected in the periphery. (H&E magnification × 20)

**Fig. 3:**
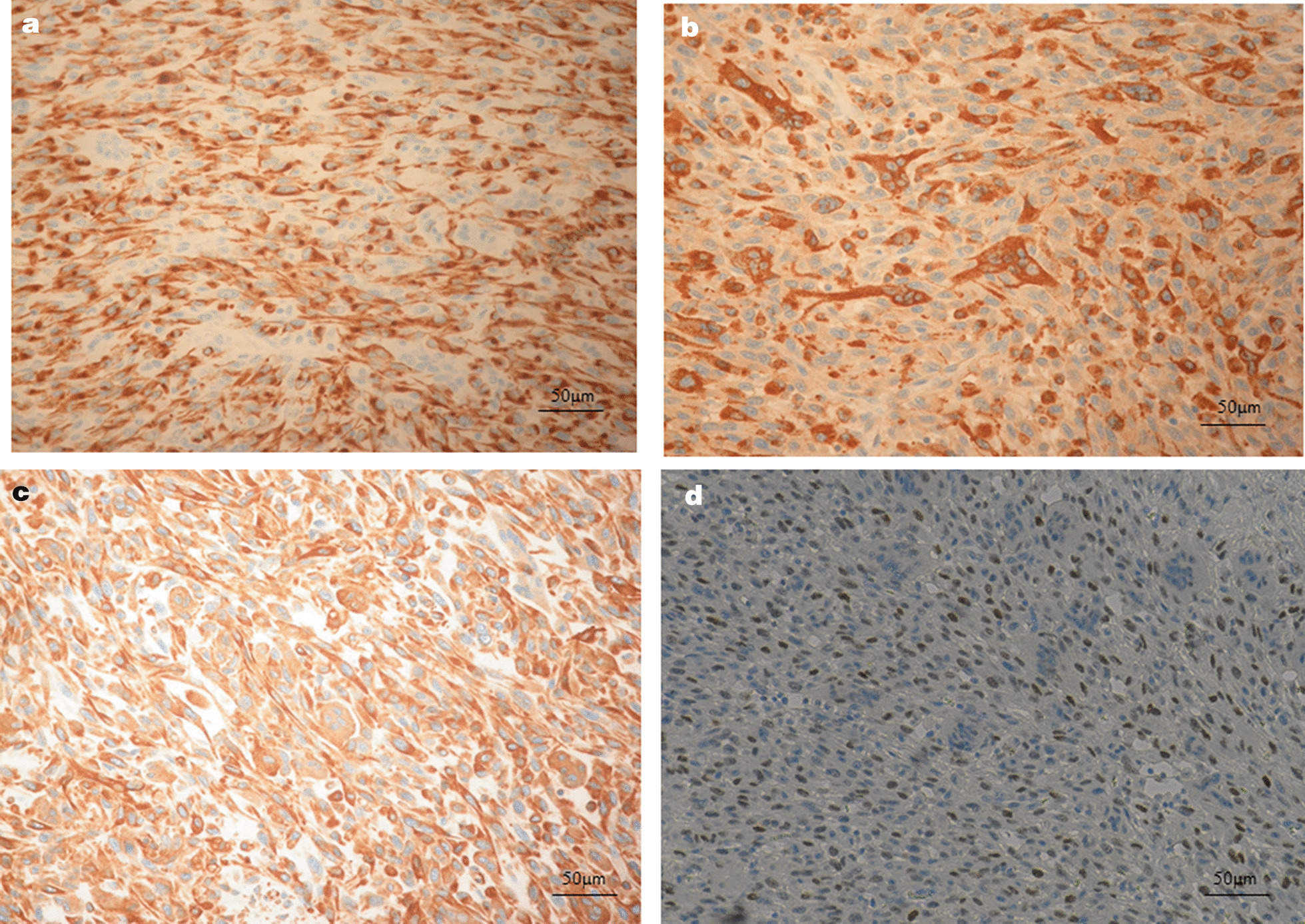
Immunoistochimical staining **a** reactivity with CKAE1/AE3 in pleomorphic neoplastic cells; Osteoclast like giant cells did not stain with CKAE1/AE3. **b** Reactivity with vimentin in pleomorphic neoplastic cells and osteoclast-like giant cells; **c** reactivity with CD68 in osteoclast-like giant cells; **d** Reactivity with p53 in pleomorphic neoplastic cells

A 67-year male was admitted to the Department of Gastroenterology for severe abdominal pain with itching, dyspnea and sensation of gastric fullness of 15 days. A laboratory examination revealed an elevated conjugate and unconjugate serum bilirubin (15.26 mg/dl and 10.62 mg/dl, respectively) and transaminase levels (ALT 208, AST 105, γGT 250 U/l). Carbohydrate antigen 19-9 levels was elevated at 93.8 U/l (vn 0–35 U/l). Abdominal computed tomography (CT) scan with contrast revealed a dilatation of the biliary tract, the bile duct and the cephalic wirsung due to the presence in the head of the pancreas of an uneven lesion with a solid vascularized component of 3 cm size. The lesion was in a close contact with the cava vein and caused local compression. There were no significantly enlarged local lymph nodes, ascites and metastases. The patient underwent to a routine pylorus preserving pancreatoduodenectomy. The intraoperative rapid frozen section disclosed a white, partly solid and partly degenerative cystic foci of 3 cm size that was detected inside of the pancreas head protruding in the duodenal lumen with ulceration of the mucous lining (Fig. 1). The report of quick-frozen sample pathological findings during surgery revealed “malignant neoplasm” with tumor-free resection margins. After careful exploration, no other lesion, the operation proceeded smoothly. Definitive histological examination revealed undifferentiated pancreatic carcinoma with highly pleomorphic neoplastic mononuclear cells and multinucleated non-neoplastic osteoclast-like giant cells (Fig. 2a). The neoplasm showed an expansive pattern infiltrating the duodenal wall with intraluminal protrusion and with ulcer-hemorrhagic phenomena. In particular, in our case we have detected the presence of very early ductal adenocarcinoma lesions associated and detected in the periphery, which attest to the ductal origin (Fig. 2b). A single posterior pancreaticoduodenal lymph nodes metastasis (1/9) was detected according to Royal College of Pathologists recommendations. Negative margins, no lymphovascular invasion and nodal metastases were given. Immunohistochemical staining revealed strongly positive pan-cytokeratin (CKAE1/AE3) in pleomorphic neoplastic cells whereas OGCs did not show reactivity with CKAE1/AE3. (Fig. 3a). OGCs was strongly immunoreactive to histiocytic markers, CD68 (Fig. 3b). Undifferentiated carcinoma component were markedly positive for vimentin (Fig. 3c). Ki67 was positive in 25% of the pleomorphic neoplastic mononuclear cells. The absence of p53 in the OGCs and its expression in pleomorphic tumor cells gave evidence of a different proliferation rate and origin of both cell populations (Fig. 3d). This morphology and immunohistochemistry-staining pattern was classified as an undifferentiated carcinoma of the pancreas with OGCs. Subsequent molecular studies were performed, Exon 2 KRAS codon 12 and 13 and BRAF codon 600 mutations were assessed by pyrosequencing. We found a mutation at codon 12 of KRAS gene (G12V) confirming the ductal cell origin of undifferentiated carcinomas and worse prognosis.

The patient was postoperatively recovered without complications. A post-operative laboratory examination revealed a reduction conjugate and unconjugated serum bilirubin (4.04 mg/dl and 2.14 mg/dl respectively) and transaminase levels (ALT 30 U/l, AST 35 U/l, γGT 49 U/l). She was discharged from hospital on post-operative day 8. The patient did not receive pre and post -operative chemotherapy Five months following surgery, the patient was generically in good condition and no recurrence or metastases was observed.

## Discussion and conclusions

Histological, cytological and survival characteristics of UCOGCs remain yet uncertain due to an uncommon tumor with a poor prognosis. Our case presented a single but important proof of undifferentiated carcinoma of pancreas with osteoclast-like giant cells.

Histologically, UCPOGC includes large multinucleated non-neoplastic osteoclast-like giant cells dispersed among pleomorphic neoplastic cells. Evidence supports that the tumor giant cells are a well-recognized entity, non-neoplastic in nature and of histiocytic origin. In the present report, multinucleated OGCs in the neoplasm were mixed among infiltrating pleomorphic mononuclear, suggesting the importance of considering the nature of the two components. OGCs no express epithelial markers (CKAE1/AE3), but have positive staining for histomonocytic markers (CD68) and lack of mitoses and proliferative activity, detected by Ki-67, suggesting to result from a fusion of mononuclear histiocytes/macrophages, chemoattracted by the tumor cells [8]. In addition, strong cytoplasmic staining p53 was observed in the cytoplasm of the pleomorphic cancer cells, yet was not detected in the OGCs. In this case, the coexistence of adenocarcinoma component and undifferentiated carcinoma component with positive staining for vimentin, suggesting that the tumor originated from pancreatic ductal cells and positivity to CKAE1/AE3 support the epithelial origin of these neoplastic components while immunoprofile of OGCs component suggested a mesenchymal origin. As such, OGCs can be regarded as a distinctive model of epithelial-mesenchymal transition but ought to be tested with further molecular analysis [9]. Actually, undifferentiated carcinoma, another uncommon pancreatic carcinoma variant, has frequent *KRAS* mutations [10, 11]. K-ras activation is an early event in the pathogenesis of pancreatic cancer and is associated with worse prognosis in pancreatic cancer and progression to undifferentiated carcinoma of the pancreas.

Molecular analyses, in particular the acquisition of KRAS status was important to clarify the nature of the cell types in undifferentiated carcinoma with osteoclast-like giant cells [12]. Molecular analysis in our case showed a mutation at codon 12 of KRAS gene (G12V), suggesting a common way to malignant transformation and a shared histogenesis arising from intraductal epithelial proliferations. In particular, in our case we have detected the presence of very early ductal adenocarcinoma lesions associated and detected in the periphery, which attest to the ductal origin. Therefore, at low magnification the neoplasm morphologically resembles the giant-cell tumor of the tendon sheath neoplasm or osteoarticular tumors. Only at high magnification, it possible to observe the cell pleomorphism and the characteristics expressed by the non-giant cell component that can take a heterogeneous morphology including spindle shaped mononuclear cells and bulky elements with a popcorn core. UCPOGC is also an aggressive tumor that usually invades adjacent organs. Moreover, lymph node involvement and distant metastasis are rarely observed in UCPOGC, such as in the presented case, no distant metastases were present at the time of surgery and on histological examination A single posterior pancreaticoduodenal lymph nodes metastasis (1/9) was detected.

We present this case to increase clinical awareness of this rare clinical entity and also review in literature the management of UC-OGC. The cases of UCP-OGC published to date are summarized in Table 1 but there are still few summary articles for the clinical, imaging and pathological features, treatment programs, prognosis and other aspects of UCP-OGC. Pancreatic UCP-OGC is more common in middle-aged and elderly patients, 86% of whom are over 50 years of age, and the average age is 63 years. Most patients are females (male: female = 8:13). The clinical symptoms are atypical and mostly manifest as upper abdominal pain, weight loss and/or anorexia. Jaundice and steatorrhea have been occurred in 25% of cases. In our case, the patient presented severe abdominal pain and jaundice.

**Table 1:** Literature review regarding patients exhibiting undifferentiated carcinoma with osteoclast-like giant cell tumors

First author	Year	Gender	Symptoms	Pathology	Pancreatic locatiom	Treatment	Survival (month)
Bedioui [13]	2004	M	Jaundice, epigastralgia	UC-OGC	Head	PD	18
Nel GA [14]	2005	M	UAP, WL, jaundice	UC-OGC + MCN	Head	PD	12
Pan [15]	2007	F	Wl, UAP, anorexia	UC-OGC + MCN	Body	PD	> 4
Manduch [16]	2009	M	WL, jaundice, pruritis	UC-OGC	Head	PD + lymph node resection	12
Rahul Mannan [17]	2010	F	Jaundice	UC-OGC	Head	PD	NA
Hur [18]	2011	F	UAP, anorexia	UC-OGC	Tail	PD	NA
Wanda [19]	2011	M	Anorexia	UC-OGC + MCN	Tail	PD	4
Kobayashi [20]	2014	F	Epigastralgia	UC-OGC	Body	PD	84
Temesgen [21]	2014	F	UAP	UC-OGC	Tail	PD	> 6
Jo [22]	2014	F	UAP, WL	UC-OGC	Body	PD	9
Sah [23]	2015	F	UAP	UC-OGC	Body and Tail	PD + lymph node resection	NA
Gao [24]	2015	F	Epigastric pain, anorexia	UC-OGC	Body and Tail	PD	120
Chiarelli [25]	2015	F	Abdominal discomfort	UC-OGC + MCN	neck and body	PD	10
Georgios [26]	2016	F	UAP, Wl, steatorrhea	UC-OGC	Head	PD	10
Saito [27]	2016	61/F	UAP	UC-OGC	Tail	PD	72
Fu [28]	2017	66/F	UAP	UC-OGC	Body	PD	10
Guo [29]	2018	65/M	UAP	UC-OGC	Head	PD	> 10
Zhang [30]	2019	57/M	Epigastric pain, WL	UC-OGC	Tail	PD	> 6
Nehmeh [31]	2019	77/M	Severe UAP	UC-OGC	Tail	PD	19
Haisam Abidc [32]	2019	62/F	Bilateral flank pain, WL	UC-OGC	Tail	PD	NA
Present case	2020	62/M	Jaudice, UAP	UC-OGC	Head	PD	> 5

*M* male, *F* female, *UAP* upper abdominal pain, *WL* weight loss, *MCN* mucinous cystic, *PD* pancreaticoduodenectomy, *NA* not available

The neoplasms are mostly located in the body and tail of the pancreas (14/21 cases) while tumors in the head or neck of the pancreas are less frequency and tend to cause dilatation of the pancreatic duct. Most of the patients in published case reports (81%) showed early recurrence and rapid progression even after complete surgical resection and died of tumor within 1 year. Although, the survival period is very large, between 4 and 120 months, which may be related to the histological heterogeneity of the tumor and the extent of the lesion at the time of discovery. In this case, the patient has no distant metastasis and after five months, postoperatively she is without evidence of recurrence or metastasis. Actually, surgical resection is the treatment of choice and remains to be demonstrated whether the response of UCPOGC to chemotherapy or radiotherapy will be efficacious although survival is very short for the patients.

Based on the present case and limited previous data, undifferentiated carcinoma of the pancreas with osteoclast-like giant cells (UCPOGC) is still a controversial subject in the literature and published data is confusing concerning histogenesis and prognosis. Further researches preferably with large cohorts are necessary to clarify the pathogenesis of the neoplasm. However, histopathological and immunohistochemical studies are the gold standard for the diagnosis of UCPOGC. Investigation of the genomic alterations in UPOGCs could help to explain the histologic diversity of variant tumor and could provide a genetic basis for prognosis and treatment.

## Data Availability

All data generated or analyzed during this study are included in this published article.

## Abbreviations

UCOGCs: Undifferentiated carcinoma with osteoclast-like giant cells
OGCs: Osteoclast-like giant cells
CT: Abdominal computed tomography
